# Effect of Polymer Encapsulation on the Mechanoluminescence of Mn^2+^-Doped CaZnOS

**DOI:** 10.3390/polym16172389

**Published:** 2024-08-23

**Authors:** Xiaohan Wu, Mengmeng Cao, Congcong Han, Jinyi Zhang, Xiangrong Li, Jieqiong Wan

**Affiliations:** College of Chemistry and Chemical Engineering, Shanghai University of Engineering Science, Shanghai 201620, China; 18253805515@139.com (X.W.); fighting5032@163.com (M.C.); 13931998329@163.com (C.H.); gumayusi914920@163.com (J.Z.); 13430564899@163.com (X.L.)

**Keywords:** mechanoluminescence, CaZnOS:Mn^2+^, polymer encapsulation, PDMS, stress sensor

## Abstract

Rare earth and transition metal ion-doped CaZnOS has garnered significant attention for its exceptional mechanoluminescence (ML) performance under mild mechanical stimuli and its capability for multicolor emissions. Since powdered phosphors are not directly usable, they require encapsulation within with polymers to create stable structures. This study investigates Mn^2+^-doped CaZnOS (CaZnOS:Mn^2+^) as the ML phosphor, optimizing its performance by varying the Mn^2+^ content, resulting in bright orange-red emissions from the d-d transitions of the Mn^2+^ activator. A quantum efficiency of 59.08% was achieved through the self-sensitization of the matrix lattice and energy transfer to the Mn^2+^ luminescent centers. The enhancement in ML due to Mn^2+^ doping is attributed to the reduced trap depth and increased trap concentration. Encapsulation with four polymers—PDMS, PU, SIL, and RTV-2—was explored to further optimize ML performance. Among these, PDMS provides the best ML output and sensitivity, owing to its slightly cross-linked structure and good triboelectric properties. The optimized CaZnOS:0.03Mn^2+^/PDMS composite, featuring excellent flexibility and recoverability, shows great potential for applications in anti-counterfeiting encryption, stress sensors, and wearable devices.

## 1. Introduction

Mechanoluminescence (ML) materials present a novel approach for directly converting mechanical energy into photon emissions without the need for external power supply. This energy can arise from sources such as friction, compression, tension, cutting, or ultrasonic waves [1]. Due to their unique properties, ML materials offer bright prospects in various applications, including stress detection and monitoring, artificial skin, and biomedical imaging. Researchers have explored several innovative uses of ML materials [2]. Patterned and colorful wind-driven devices have been fabricated by embedding ZnS:Cu^+^/Mn^2+^ ML particles in a polydimethylsiloxane (PDMS) composite, demonstrating their potential for use in practical displays and lighting systems [3]. Temperature and pressure bimodal sensing, with promising uses in tactile sensing and user-interactive technologies, has been achieved by combing ZnS-CaZnOS ML hybrids and PEDOT [4]. Furthermore, dynamic mechanical actions can be visualized with a near-distance sensor that integrates a ZnS:Cu^+^/PDMS film with a CMOS detector, achieving high sensitivity with a detection limit down to kPa, a high spatial resolution of 254 dpi, and a fast response time of 3.3 ms [5]. The key to fabricating highly sensitive ML sensors lies in adopting devices with a superior ML response under faint mechanical stimuli. Numerous efforts have been focused on developing novel ML materials with strong ML, including SrAl_2_O_4_:Eu^2+^, ZnS:Cu^+^, Mn^2+^, CaZnOS:Mn^2+^, rare earth ions, and LiNbO_3_:Pr^3+^ [6]. Among these materials, rare earth and transition metal ion-doped CaZnOS has garnered significant attention for its high ML intensity and multicolor emissions. One of the advantages is the unique asymmetric layered crystal structure of CaZnOS, which facilitates strong ML responses even to weak mechanical stimuli, making it an ideal candidate for highly sensitive stress sensors. In addition, the two available doping sites (Ca^2+^ and Zn^2+^) of CaZnOS lattice allows the doping of a wide range of lanthanide rare earth and transition metal ions [7]. On account of that, it is convenient to obtain multicolor ML emissions in CaZnOS-based materials, spanning from near ultraviolet to infrared light [8]. Among the various dopant activators, Mn^2+^-doped CaZnOS exhibits the best ML intensity, emitting bright orange-red light visible to the naked eye even in daylight conditions, and has thus received widespread attention [9].

To optimize the performance of ML materials and devices, various strategies have been employed. These include designing heterostructures and implementing co-doping or cation substitution [10,11,12]. These methods have significantly improved the ML performance. Beyond merely increasing the ML intensity of the material itself, the interactions between ML powder and the polymer significantly impact the ultimate luminescence intensity of ML devices. Polymer chains transmit external forces to phosphor particles, triggering the ML and significantly impacting the device’s overall performance. By designing different polymer layers, such as a triple-sandwich structure containing ZnS:Cu^+^, Mn^2+^/PVDF/EVA/PET layers, the ML intensity improved by approximately 85% compared to the simpler ZnS:Cu^+^, Mn^2+^/EVA/PET structure [13]. Song et al. prepared a polymeric film of ZnS:Cu^+^, Mn^2+^/PDMS@Al_2_O_3_, with the addition of SiO_2_ nanoparticles to aid in concentrating stress conduction, thereby achieving strong ML [14]. The polymer matrix influences the final ML intensity of ML devices through mechanisms, including force transmission, stress concentration, flexibility, surface interaction, and protection of the ML phosphor particles [15]. As a result, selecting an appropriate polymer and optimizing its interaction with ML phosphors are crucial for enhancing the performance of ML devices.

In this study, we selected CaZnOS:Mn^2+^ as the ML phosphor, and four commonly used polymers—polydimethylsiloxane (PDMS), polyurethane (PU), silicone gel (SIL), and hydrophobic room-temperature vulcanized silicone rubber (RTV-2)—as matrices to investigate the effect of polymer encapsulation on the ML performance of the devices. Firstly, we optimized the ML performance of CaZnOS:Mn^2+^ powders by screening different Mn^2+^ concentrations. We found that CaZnOS:0.01Mn^2+^ exhibited the highest photoluminescence (PL) with a quantum efficiency of 59.08%, while CaZnOS:0.03Mn^2+^ showed the highest ML, with very bright orange-red ML observable when slightly rubbing with a glass rod. These four polymers were then used as encapsulation matrices to explore their effects on ML intensity of CaZnOS:0.03Mn^2+^. Despite CaZnOS:Mn^2+^ being a typical ML material, previous studies have not investigated the influence of a polymer matrix on its ML intensity and sensitivity under faint stimulation. Most studies have primarily focused on rigid CaZnOS:Mn^2+^/epoxy resin composites subjected to substantial pressures (1000~5000 N) [9,16]. Herein, we investigated the response characteristics of the four types of CaZnOS:0.03Mn^2+^/polymer composites under small stresses (5~27.5 N) and found that CaZnOS:0.03Mn^2+^/PDMS exhibited the highest ML intensity and sensitivity. The mechanism behind enhanced ML performance through PDMS encapsulation, which involves piezoelectricity- and triboelectricity-induced ML, was explored. After encapsulation in PDMS, a CaZnOS:0.03Mn^2+^-based composite film demonstrated bright orange-red ML even under very slight stimulation, achieving an ML intensity comparable to that of commercially available ZnS:Cu^+^. The fabricated composite film exhibited excellent flexibility and recoverability, showing great potential in flexible anti-counterfeiting, stress sensing, and visualization.

## 2. Results and Discussion

**Figure 1:**
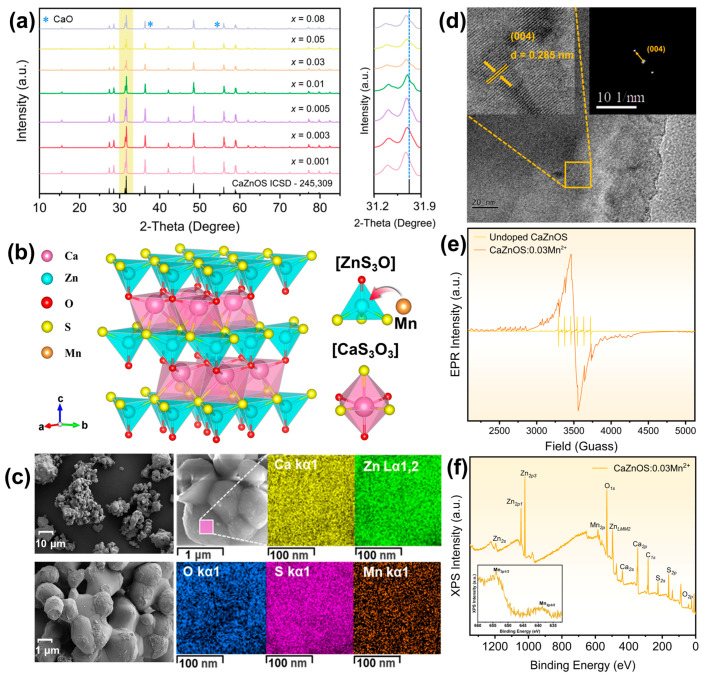
(**a**) Left: X-ray diffraction (XRD) patterns; Right: a selected region (31.2~31.9°) of the XRD patterns of CaZnOS:*x*Mn^2+^ (0.001 ≤ *x* ≤ 0.08) samples. (**b**) Schematic representation of the layered crystal structure of CaZnOS and the Mn^2+^ doping site. (**c**) Scanning electron microscope (SEM) images and elemental mapping of CaZnOS:0.05Mn^2+^. (**d**) High resolution transmission electron microscope (HRTEM) image and selected area electron diffraction (SAED) pattern of CaZnOS:0.03Mn^2+^. (**e**) Electron paramagnetic resonance (EPR) spectra of CaZnOS and CaZnOS:0.03Mn^2+^. (**f**) X-ray photoelectron spectroscopy (XPS) spectrum of CaZnOS:0.03Mn^2+^.

The Mn^2+^-doped CaZnOS samples (abbreviated as CaZnOS:*x*Mn^2+^, 0.001 ≤ *x* ≤ 0.08) were synthesized using the conventional high-temperature solid-state method. The synthetic details are provided in the Supplementary Materials. The X-ray diffraction (XRD) patterns of these as-prepared CaZnOS:*x*Mn^2+^ samples are presented in Figure 1a. The patterns of fully concentrated samples closely match the CaZnOS standard card (ICSD#24-5309) [7]. Traces of CaO impurity, resulting from the decomposition of raw material CaCO_3_, have been consistently observed alongside the CaZnOS products prepared using the solid-state method [17,18]. All the as-prepared CaZnOS:Mn^2+^ samples exhibit excellent crystallinity. The crystal structure of CaZnOS is shown in Figure 1b. CaZnOS possesses a non-centrosymmetric structure and belongs to the P6_3_*mc* space group, comprising two puckered [Zn_3_O] tetrahedral layers and [CaO_3_S_3_] octahedral layers [19]. This unique topological arrangement grants doped CaZnOS exceptional ML performance. Due to the similarity in ionic radius, Mn^2+^ (r = 0.66 Å) ions tend to occupy Zn^2+^ (r = 0.60 Å) sites over Ca^2+^ (r = 1.00 Å) sites [20]. As depicted in the right column of Figure 1a, increasing Mn^2+^ doping leads to a shift in the XRD peaks towards smaller angles, indicating that a lattice expansion was introduced after Mn^2+^ doping. Since the ion radius of Mn^2+^ is slightly larger than that of Zn^2+^, Mn^2+^ substitution causes lattice distortion and enlarges the unit cell, consistently with the observed shift result in the XRD peaks. This result suggests the successful incorporation of Mn^2+^ into the CaZnOS host matrix. The morphology of the CaZnOS:0.05Mn^2+^ sample is illustrated in the inset of Figure 1c. CaZnOS:Mn^2+^ exhibits micron-sized particles with a smooth, polygonal shape, characteristic of products obtained through high-temperature solid-state calcination. The elemental mapping of a selected particle among the CaZnOS:0.05Mn^2+^ powders is presented in Figure 1c, demonstrating uniform distribution of Ca, Zn, O, S, and Mn. The corresponding energy dispersive spectroscopy (EDS) result is displayed in Figure S1, showing a Ca:Zn:Mn:S molar ratio of 1:0.997:0.052:0.966, which closely aligns with the theoretical composition of 1:0.95:0.05:1. Figure 1d presents the HRTEM result of the CaZnOS:0.03Mn^2+^ powder, showing the view along the [004] projection. The high resolution transmission electron microscope (HRTEM) image reveals distinct lattice fringes with interplanar d-spacings of 0.285 nm, corresponding to the [004] crystal faces of hexagonal CaZnOS:Mn^2+^. The selected area electron diffraction (SAED) pattern confirms the single-crystalline nature of CaZnOS:0.03Mn^2+^. To confirm the presence of Mn^2+^, an electron paramagnetic resonance (EPR) analysis of undoped CaZnOS and CaZnOS:0.03Mn^2+^ was conducted. As shown in Figure 1e, a strong signal originating from Mn is detectable after doping [21,22]. Additionally, a chemical element analysis of the CaZnOS:0.03Mn^2+^ sample was conducted using an X-ray photoelectron spectroscopy (XPS) test, with the result shown in Figure 1f. Signals from Ca, Zn, O, and S are observable in the range of 0~1250 eV. Specifically, the characteristic peaks located at 637 and 653 eV correspond to ${\mathrm{Mn}}_{2p1/2}$ and ${\mathrm{Mn}}_{2p3/2}$, respectively, demonstrating the successful doping of Mn^2+^ [23].

**Figure 2:**
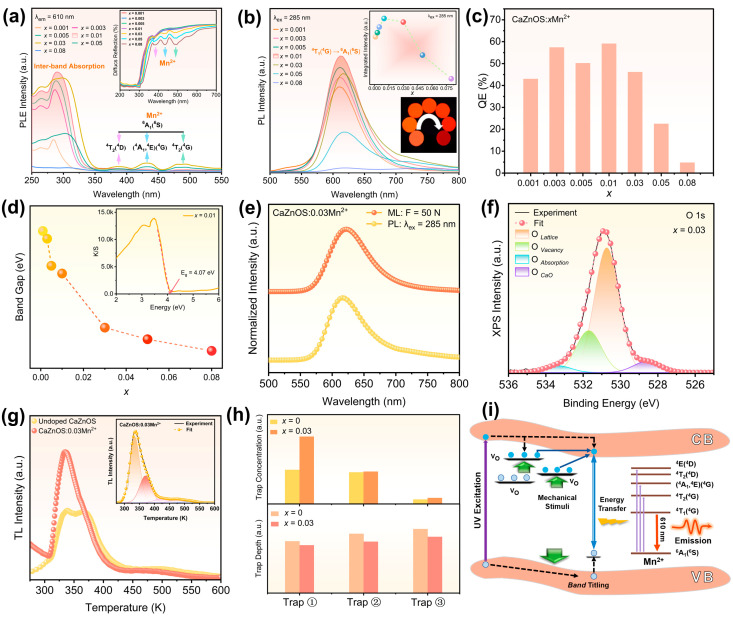
(**a**) Photoluminescence excitation (PLE) spectra (λ_em_ = 610 nm), with the inset showing the corresponding diffuse reflection spectra. (**b**) Photoluminescence (PL) spectra (λ_ex_ = 285 nm), with the upper inset showing the variation of integrated PL intensity with varied Mn^2+^ concentration (*x*) and the lower inset showing the corresponding photos taken under the irradiation of 275 nm UV light. (**c**) Quantum efficiency. (**d**) The band gap, with the inset showing the band gap diagram of sample CaZnOS:0.01Mn^2+^, for CaZnOS:*x*Mn^2+^ (0.001 ≤ *x* ≤ 0.08). (**e**) Mechanoluminescence (ML) and PL spectra of CaZnOS:0.03Mn^2+^. (**f**) Peak fitting result of the O1s XPS spectrum of CaZnOS:0.03Mn^2+^. (**g**) Thermoluminescence (TL) spectra of the CaZnOS matrix and CaZnOS:0.03Mn^2+^, with the inset showing the fitting result of the TL spectrum of CaZnOS:0.03Mn^2+^. (**h**) Trap depth and concentration of CaZnOS and CaZnOS:0.03Mn^2+^. (**i**) Schematic diagram of the ML processes involving CaZnOS:Mn^2+^.

Before delving into exploring the ML characteristics, we initially investigated the photoluminescence (PL) properties of CaZnOS:Mn^2+^. Figure 2a illustrates the photoluminescence excitation (PLE) spectra of the series of CaZnOS:*x*Mn^2+^ (0.001 ≤ *x* ≤ 0.08) samples. The inset shows the corresponding diffuse reflectance spectra (DRS). The absorption peaks in the excitation spectra are consistent with the DRS results. Figure 2b displays the emission spectra (λ_ex_ = 285 nm) of the CaZnOS:Mn^2+^ samples. The broad-band emissions covering 550~750 nm arises from electron transitions of Mn^2+^ between the excited state of ^4^T_1_(^4^G) and the ground state of ^6^A_1_(^6^S) [19]. The quantum efficiency of CaZnOS:*x*Mn^2+^ samples, presented in Figure 2c, shows the CaZnOS:0.01Mn^2+^ sample reaching 59.08% (Figure S2), indicating the excellent PL performance of the prepared CaZnOS:Mn^2+^ samples. The PLE spectra (Figure 2a) encompass the dominant absorption in the ultraviolet region (250~300 nm), as well as three significantly weaker absorption peaks at 386 nm, 437 nm, and 496 nm. The optical band gap of CaZnOS:Mn^2+^, calculated using the Kubelka Munk equation and shown in Figure 2d, is in the range of 3.87~4.18 eV, aligning with reported values of 3.5~4.16 eV [24]. These results confirm the semiconductor nature of CaZnOS. Hence, the strong ultraviolet absorption originates from the CaZnOS host matrix, due to electron transitions from the valence band maximum to the conduction band minimum. The three extremely weak absorption peaks arise from the characteristic electron transitions of the doped Mn^2+^ ions, specifically from the ^6^A_1_(^6^S) ground state to the excited states of ^4^T_2_(^4^D), (^4^A_1_,^4^E)(^4^G), and ^4^T_2_(^4^G). The weak absorption of Mn^2+^ is attributed to the spin/orbital-forbidden d–d transitions in Mn^2+^ [19]. Despite the forbidden nature of these transitions, CaZnOS:Mn^2+^ exhibits bright orange-red emissions upon irradiation of a 5 W, 275 nm flashlight and high quantum efficiency, as shown in the inset of Figure 2b,c. These efficient emissions are attributed to the lattice self-sensitization effect in CaZnOS:Mn^2+^. The host lattice absorbs the excitation ultraviolet light and subsequently transfers the energy to Mn^2+^ centers, which triggers the emissions. By utilizing a broader absorption spectrum of the excitation source, beyond the intrinsic spin/orbital-forbidden d–d absorption of Mn^2+^, as depicted in Figure 2a, CaZnOS:Mn^2+^ achieves enhanced light harvesting [25]. As Mn^2+^ doping increases, the emission intensity initially increases, reaching a peak at *x* = 0.01, and then decreases due to concentration quenching when Mn^2+^ doping exceeds 1 mol%. Additionally, increasing Mn^2+^ doping leads to an 18 nm redshift in the emission spectra of CaZnOS:Mn^2+^ samples (Figure S3), attributed to the reduced distance between adjacent Mn^2+^ centers at higher doping levels [24].

**Figure 3:**
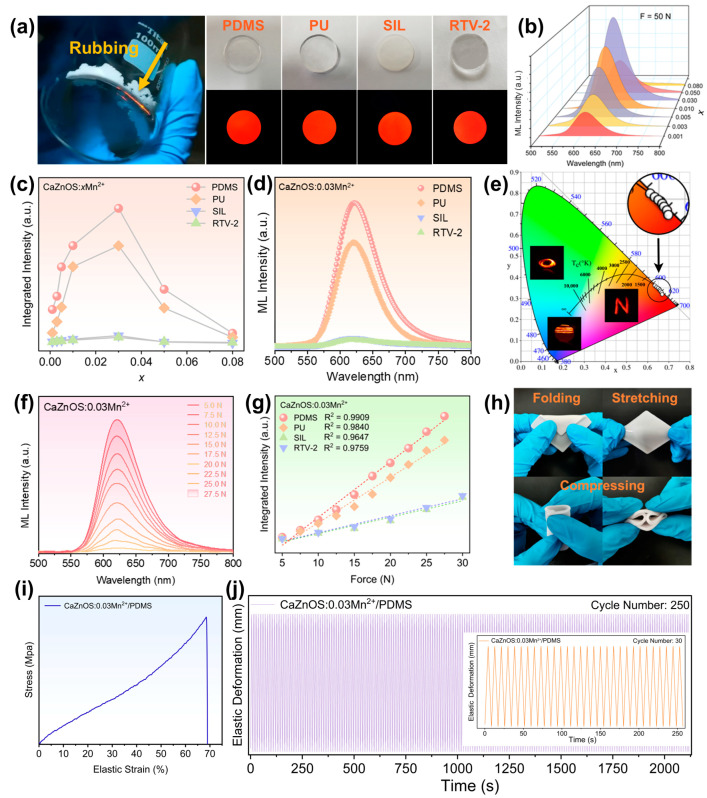
(**a**) (**left**) Video screenshots of rubbing the CaZnOS:0.03Mn^2+^ powder with a glass rod; (**right**) Photos of four polymer matrices under daylight (**upper**) and the encapsulated composite films under 275 nm light irradiation (**below**). (**b**) ML spectra of CaZnOS:*x*Mn^2+^/PDMS films driven by 50 N. (**c**) Integrated ML intensity of four types of encapsulated films with varied Mn^2+^ concentration driven by 50 N. (**d**) ML spectra of four polymers encapsulating CaZnOS:0.03Mn^2+^ driven by 50 N (**e**) CIE coordinates of PDMS encapsulating CaZnOS:*x*Mn^2+^. (**f**) ML spectra of CaZnOS:0.03Mn^2+^/PDMS subjected to varying dynamic loads. (**g**) Quantitative relationship between the integrated ML intensity and applied load for four CaZnOS:0.03Mn^2+^ encapsulated polymers. (**h**) Photos of CaZnOS:0.03Mn^2+^/PDMS flexible film upon folding, stretching, and compressing under daylight. (**i**) Stress–strain curve and (**j**) mechanical strength of the CaZnOS:0.03Mn^2+^/PDMS film during 250 repeated stretching and releasing cycles, with an inset providing a detailed view of the elastic deformation over 30 cycles.

The prepared CaZnOS:Mn^2+^ powders exhibit visibly bright orange-red ML when subjected to friction with a glass rod, similarly to their PL, as shown in Video S1 and Figure 3a (left). The ML spectrum, along with the PL spectrum, is shown in Figure 2e. Due to the strong energy transfer from the CaZnOS host lattice to the Mn^2+^ dopant activators, it is speculated the intrinsic traps of the matrix greatly impact the ML performance. Oxygen vacancies are crucial traps that determine the ML performance of CaZnOS-based materials [26,27]. X-ray photoelectron spectroscopy (XPS) and thermoluminescence (TL) tests were performed to characterize the oxygen vacancies in CaZnOS:Mn^2+^. The fitting results of the O1s signal are displayed in Figure 2f. The dividing four peaks are from the lattice O in CaO impurity (528.6 eV) [28,29], lattice oxygen (530.7 eV), oxygen vacancy (531.8 eV), and adsorption oxygen (533.4 eV) in CaZnOS [30,31]. The XPS results confirmed the presence of oxygen vacancies. Furthermore, the TL results of the CaZnOS matrix and CaZnOS:0.03Mn^2+^ are illustrated in Figure 2g. The peak fitting results of the TL signal of CaZnOS:0.03Mn^2+^ are depicted in the inset of Figure 2g and Table S1. The TL spectra were analyzed using Chen’s kinetic equation (Equation (1)) to obtain specific trap depth of ①, ②, and ③:

(1) $$E=3.5{(\mathrm{kT}}_{m}^{2}/\omega )-2kT_{m}$$

where *E* represents the trap depth, *k* is the Boltzmann constant (8.617 × 10^−5^ eV), *T_m_* is the peak temperature (K), and *ω* is the half-width at half-maximum [32,33].

The results show that the trap depths of ①, ②, and ③ are 0.807 eV, 0.867 eV, and 0.949 eV, respectively. The TL analysis of the CaZnOS matrix is presented in Figure S4 and Table S1. After Mn^2+^ doping, the trap depth was reduced, and the trap concentration increased significantly, as shown in Figure 2h. This increase in trap concentration allows for the accommodation and release of more carriers, thereby enhancing the ML intensity. Furthermore, the decrease in trap depth induced by Mn^2+^ doping makes it easier for charge carriers to be released in response to external stress stimuli. This significantly improves the mechanical sensitivity response while enhancing the ML intensity. Based on the above analysis, the proposed mechanism involving the ML process in CaZnOS:Mn^2+^ is illustrated in Figure 2i. Upon excitation with UV light, electrons are promoted from the valence band to the conduction band, where the free carriers then transport them. Some of these carriers recombine with holes via non-radiative transitions, transferring energy to Mn^2+^ activators, resulting in PL emissions. Meanwhile, some photon-generated carriers and holes are captured and remain within the oxygen and cation vacancies. When external stress is applied, the CaZnOS:Mn^2+^, which features a non-centrosymmetric crystal structure and a high piezoelectric coefficient (d_33_ = 38 pm V^−1^) [7], undergoes lattice deformation and generates an inner piezoelectric field. This lattice deformation leads to the tilting of the valence and conduction bands, which reduces the depth of the trap states and alleviates the energy barrier [9]. The piezoelectric field further aids in the release of trapped carriers and holes into the conduction and valence bands. These released free carriers then recombine through nonradiative transitions and transfer energy to the Mn^2+^ activators, enabling ML emissions via d-d transitions of Mn^2+^.The doping of Mn^2+^ additionally reduces the trap depth and increases the trap concentration, thereby enhancing the ML intensity, which reaches its maximum when the Mn^2+^ doping content is at 3%. The ML repeatability and degradation performance of CaZnOS:0.03Mn^2+^ were assessed, with the methods detailed in the Supplementary Materials and results displayed in Figure S5. CaZnOS:Mn^2+^ primarily exhibits characteristics of a trap-controlled ML material [6]. This indicates that the energy absorbed through ultraviolet light irradiation and stored in trap states is gradually depleted through ML emissions. The degradation test results of CaZnOS:0.03Mn^2+^ align with this characteristic. As demonstrated in Figure S5a, by rapidly applying and releasing heavy pressure—from 0 to 5000 and back to 0 N within 15 s—the ML light gradually weakens and becomes essentially undetectable after 5 cycles. Nonetheless, CaZnOS:Mn^2+^ is a non-destructive ML material. Once replenished with ultraviolet light, its ML intensity can be restored to the original level, as shown in Figure S5b. This indicates that the reproducibility of CaZnOS:Mn^2+^ is highly favorable for practical applications.

To fabricate sensors and devices based on ML, the ML powders are typically encapsulated within a polymer matrix to form various shapes and microstructures. To create highly sensitive devices utilizing CaZnOS:Mn^2+^ and explore the effect of a polymer matrix on ML intensity, four polymers—PDMS, PU, SIL, and RTV-2—were employed to produce encapsulated films. Their ML performance was subsequently studied. Photos of these polymer matrices under daylight and the corresponding encapsulated composite films under the radiation of a 5 W, 275 nm flashlight are presented in Figure 3a (right). Bright orange-red light can be observed when sliding on the film, and the corresponding ML spectra were collected using a custom-built device (Figure S6). The ML spectra of fully concentrated CaZnOS:Mn^2+^ encapsulated films in PDMS, PU, SIL, and RTV-2, subjected to a 50 N stimulus, are shown in Figure 3b and Figure S7a–c. Similarly to the PL spectra of CaZnOS:Mn^2+^ powders, these films exhibit broad-band emissions from the Mn^2+^ dopant with a redshift (Figure S8) as Mn^2+^ concentration increases. The integrated ML intensity of the fabricated films is displayed in Figure 3c. Notably, the CaZnOS:Mn^2+^/PDMS and CaZnOS:Mn^2+^/PU films outperform the CaZnOS:Mn^2+^/SIL and CaZnOS:Mn^2+^/RTV-2 films. Figure 3d shows the ML spectra of the four CaZnOS:0.03Mn^2+^ encapsulated films under a 50 N stimulus, with PDMS encapsulation yielding the best ML performance. The optimized CaZnOS:0.03Mn^2+^/PDMS demonstrated a comparable ML output to commercially available ZnS:Cu^+^/PDMS under a 10 N stimulus, as shown in Figure S9. The chromaticity coordinates and photos of the ML of fully concentration CaZnOS:Mn^2+^/PDMS films is displayed in Figure 3e, revealing a bright and stable orange-red luminescence clustered within a narrow range. The sensitivity of four CaZnOS:0.03Mn^2+^ encapsulated films were investigated. The ML spectra with external stimuli ranging from 5 to 27.5 N are depicted in Figure 3f and Figure S10a–c. Figure 3g plots the integrated ML intensity as a function of applied stress, revealing a linear dependence of the emission intensity on the applied force. The CaZnOS:0.03Mn^2+^/PDMS film exhibited the best sensitivity. These results demonstrate that PDMS encapsulation provides the best ML intensity and sensitivity for CaZnOS:Mn^2+^. Additionally, the CaZnOS:0.03Mn^2+^/PDMS film exhibits excellent flexibility and durability, as shown in Figure 3h. The film resists damage even when subjected to folding, stretching, or compressing. Its tensile strength and durability were further assessed, as shown in Figure 3i,j. The film can endure a maximum elastic strain of 68.98% at a tensile speed of 500 mm min^−1^, demonstrating its toughness and resistance to damage. Throughout 250 stretching and releasing cycles under a 30% strain, the film maintains stable elastic deformation and exhibits good recovery after testing. Its flexibility and recoverability make it well suited for a broad range of applications, including flexible stress sensing, imaging, and wearable devices.

**Figure 4:**
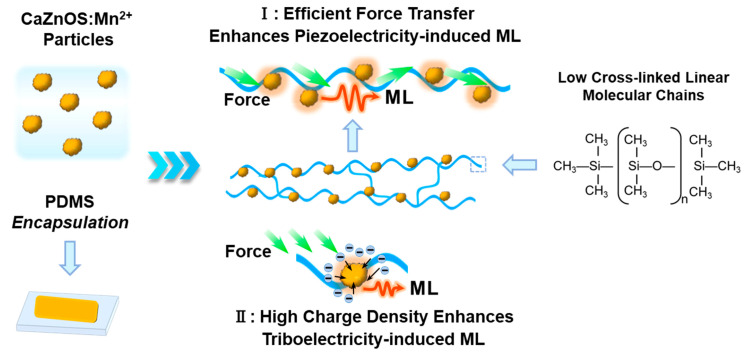
Schematic diagram of PDMS encapsulation-induced ML enhancement.

Figure 3c,d,g vividly demonstrate that polymer encapsulation markedly boosts the ML output of phosphor in composites. The ML phenomenon in ML phosphor/polymer composite is driven by piezoelectric and triboelectric effects. Piezoelectric-induced ML primarily involves trap-controlled ML materials where the ML phosphor itself exhibits piezoelectric properties. Upon the application of external force, repeated mechanical stimuli induce movement or deformation of the polymer chains. These chains transmit the force to the ML phosphor particles, causing the deformation of the phosphor lattice. This deformation facilitates charge separation within the lattice and reduces the energy barriers for trapped electrons, allowing their release into the conduction band. These electrons then migrate to the luminescent centers, culminating in ML emissions [7,34,35]. On the other hand, triboelectric effect-induced ML, which is prominent in self-recoverable materials like ZnS:Cu^+^ and ZnS:Mn^2+^, arises from contact electrification at the phosphor–polymer interface upon friction. The electrons generated by this contact electrification are transferred to the ML phosphor and subsequently conveyed to the luminescent centers, initiating ML emissions [36,37,38]. CaZnOS:Mn^2+^ displays both trap-controlled and non-trap-controlled ML characteristics, with the former playing a more substantial role in ML intensity [6]. This demonstrates that CaZnOS:Mn^2+^ employs both piezoelectric and triboelectric effects, with the piezoelectric mechanism being predominant. In terms of piezoelectric-induced ML, the movement and deformation upon external force are crucial, as they determine the degree of lattice deformation and thus the strength of the local piezoelectric field. ML performance variations across different polymers—PDMS, PU, SIL, and RTV-2—are primarily attributed to their distinct mechanical properties. PDMS, with a linear or slightly cross-linked [(CH_3_)_2_SiO] structure, exhibits excellent flexibility and extensibility. Similarly, PU, customizable in terms of cross-linking density, showed high flexibility in our experiments, indicative of low cross-linking density. In contrast, SIL and RTV-2, which are composed of highly cross-linked 3D [(CH_3_)_2_SiO] network structures, are generally harder and more brittle compared to PDMS and PU. Conversely, the higher cross-linking densities in SIL and RTV-2 result in significantly weaker luminescence due to restricted molecular mobility, diminishing their ability to effectively transmit stress to luminescent particles. For triboelectric-induced ML, its performance depends on the generated triboelectricity and the efficiency of charge transfer between materials. It has been demonstrated in other recoverable ML materials that PDMS encapsulation generates a higher triboelectric charge density upon friction compared to PU, thus leading to greater triboelectric-induced ML intensity [37,39,40,41]. Consequently, the unique linear structure and excellent triboelectric properties of PDMS contribute significantly to both piezoelectric-induced and triboelectric-induced ML, achieving the highest ML intensity, as illustrated in Figure 4.

**Figure 5:**
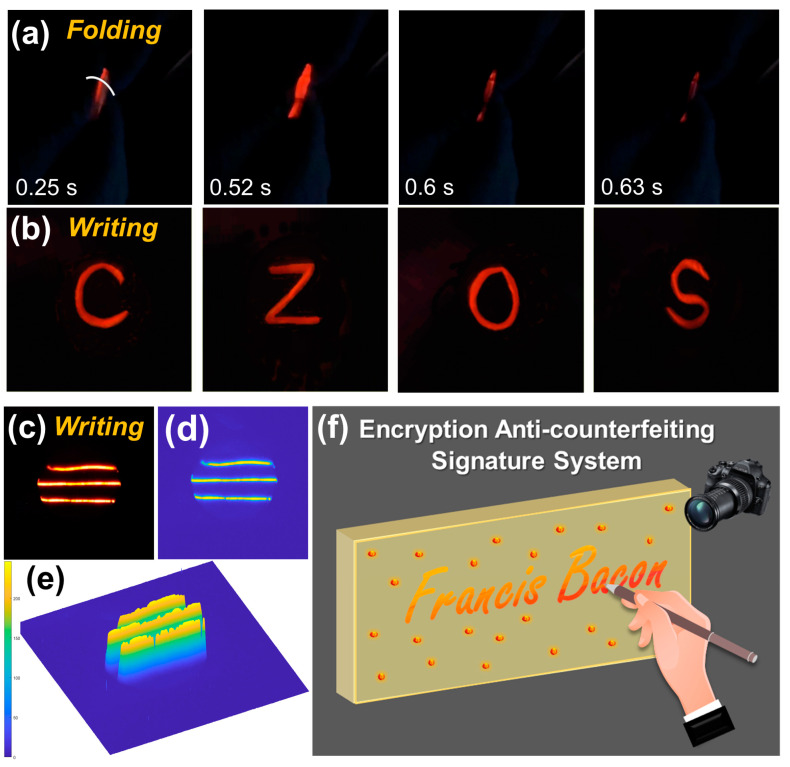
(**a**) Video screenshots of folding. (**b**) Photos of writing four element symbols on the CaZnOS:0.03Mn^2+^/PDMS flexible composite film. (**c**) Photo of the written lines. (**d**) 2D and (**e**) 3D visualization of relative force distribution extracted from (**c**) based on the gray scale value. (**f**) Schematic diagram of anti-counterfeiting signature system of CaZnOS:0.03Mn^2+^/PDMS.

The CaZnOS/PDMS film exhibited excellent flexibility and recoverability. When the CaZnOS:Mn^2+^/PDMS film is gently folded, it displays bright orange-red ML emissions (Video S2), with screenshots from the video shown in Figure 5a. To demonstrate its high ML intensity, four element symbols—Ca, Zn, O, and S—were written on the film, and corresponding photos were recorded, revealing bright and clear markings, as shown in Figure 5b. Furthermore, three lines were written on the flexible film, as demonstrated in Figure 5c. By extracting the distribution of these traces, the stress intensity information at each position was obtained, with the 2D and 3D distribution information shown in Figure 5d and Figure 5e, respectively. The map reveals that even small variations in writing are instantly reflected in the light intensity. As a potential application, an encryption anti-counterfeiting signature system can be designed using the optimized CaZnOS:0.03Mn^2+^/PDMS composite film, as demonstrated in Figure 5f. The system captures handwriting, including the coordinates and pressure of each stroke, by converting them into optical signals through the composite film. These signals are captured by a CMOS sensor, converted into electrical signals, and then stored. Furthermore, this formulation can be utilized to create various shapes using 3D printing and other molding technologies, enabling applications such as stress sensors, display technologies, and wearable devices.

## 3. Conclusions

A series of CaZnOS:*x*Mn^2+^ samples with varying Mn^2+^ doping concentrations (0.001 ≤ *x* ≤ 0.08) were synthesized to optimize the ML performance. The as-prepared powders exhibit bright orange-red PL and ML emissions, arising from d-d transitions of the Mn^2+^ activators. The excitation process includes the self-sensitization of the matrix lattice and energy transfer to the Mn^2+^ dopants, which activate the originally weak Mn^2+^ emissions. A quantum efficiency as high as 59.08% was thereby achieved. Oxygen vacancies, which were confirmed by XPS and TL spectra, play a crucial role in the ML of the CaZnOS:Mn^2+^. Mn^2+^ doping reduces trap depth and increases trap concentration, with the optimal doping for ML intensity found to be CaZnOS:0.03Mn^2+^. Moreover, the screening of polymer matrices PDMS, PU, SIL, and RTV-2 revealed that PDMS encapsulation provide the best force transmission capability. The slightly cross-linked linear structure of PDMS imposes minimal restrictions on the motion of molecular chains, leading to the significant deformation of the CaZnOS:Mn^2+^ lattice. This deformation generates a piezoelectric field that promotes the recombination of the trapped carriers, enhancing the piezoelectricity-induced ML. Additionally, PDMS’s ability to generate triboelectric charges further boosts the triboelectricity-induced ML. Consequently, the CaZnOS:0.03Mn^2+^/PDMS composite exhibits outstanding ML output and stress sensitivity, demonstrating an ML intensity that is comparable to the commercial ZnS:Cu^+^/PDMS. This composite also shows excellent flexibility and recoverability, making it highly suitable for applications in anti-counterfeiting encryption, micro-force real-time sensors, and wearable devices.

## Data Availability

The original contributions presented in the study are included in the article/Supplementary Materials. Further inquiries can be directed to the corresponding author.

## Supplementary Materials

The following supporting information can be downloaded at: https://www.mdpi.com/article/10.3390/polym16172389/s1, Figure S1: EDS spectrum and elemental composition of CaZnOS:0.05Mn^2+^. Figure S2: The quantum efficiency of CaZnOS:0.01Mn^2+^. Figure S3: (a) Normalized PL spectra; (b) CIE coordinates of CaZnOS:*x*Mn^2+^ with varied Mn^2+^ concentration. Figure S4: Fitting result of the TL spectrum of CaZnOS matrix. Table S1: Fitting result parameters of the TL spectra of CaZnOS:0.03Mn^2+^ and CaZnOS matrix. Figure S5: (a) ML degradability and (b) repeatability of CaZnOS:0.03Mn^2+^/epoxy resin composite. (c) The photo of the testing sample: CaZnOS:0.03Mn^2+^/epoxy resin composite block. Figure S6: The custom-built system for collecting mechanoluminescence spectrum. Figure S7: ML spectra of CaZnOS:*x*Mn^2+^/PU, CaZnOS:*x*Mn^2+^/SIL, and CaZnOS:*x*Mn^2+^/RTV-2 composite devices. Figure S8: Normalized ML spectra of CaZnOS:*x*Mn^2+^ with varied Mn^2+^ concentration. Figure S9: Comparison of ML spectra between CaZnOS:0.03Mn^2+^ and commercially available ZnS:Cu^+^ driven by 10 N. Figure S10: ML spectra of CaZnOS:0.03Mn^2+^/PU, CaZnOS:0.03Mn^2+^/SIL, and CaZnOS:0.03Mn^2+^/RTV-2 subjected to varying dynamic loads. Video S1: Rubbing the CaZnOS:0.03Mn^2+^ powder with a glass rod. Video S2: Folding the CaZnOS:0.03Mn^2+^/PDMS flexible film.
