# Supplementary material for: Effect of Polymer Encapsulation on the Mechanoluminescence of Mn2+-Doped CaZnOS
Source: Polymers (Basel). 2024 Aug 23;16(17):2389. doi: 10.3390/polym16172389 (PMC11397280; doi:10.3390/polym16172389)
Supplement: Supplementary file 1 [file polymers-16-02389-s001.zip › Supplementary Materials.pdf]

Supplementary Materials:

Effect of Polymer Encapsulation on the Mechanoluminescence of Mn2+-Doped CaZnOS

Xiaohan Wu, Mengmeng Cao, Congcong Han, Jinyi Zhang, Xiangrong Li, Jieqiong Wan *

Contents

1. Experimental Section

   1.1 Materials

   1.2 Preparation of CaZnOS:Mn^{2+} powder

   1.3 Preparation of CaZnOS:Mn^{2+}/polymer composite devices

   1.4 Preparation of CaZnOS:Mn^{2+}/polymer epoxy resin block

2. Characterization

3. Figures and Tables

Figure S1: EDS spectrum and elemental composition of CaZnOS:0.05Mn^{2+}.

Figure S2: The quantum efficiency of CaZnOS:0.01Mn^{2+}.

Figure S3: (a) Normalized PL spectra; (b) CIE coordinates of CaZnOS:$x$$Mn^{2+}$ with varied Mn^{2+} concentration.

Figure S4: Fitting result of the TL spectrum of CaZnOS matrix.

Table S1: Fitting result parameters of the TL spectra of CaZnOS:0.03Mn^{2+} and CaZnOS matrix.

Figure S5. (a) ML degradability and (b) repeatability of CaZnOS:0.03Mn^{2+}/epoxy resin composite. (c) The photo of the testing sample: CaZnOS:0.03Mn^{2+}/epoxy resin composite block.

Figure S6: The custom-built system for collecting mechanoluminescence spectrum.

Figure S7: ML spectra of CaZnOS:$x$$Mn^{2+}$/PU, CaZnOS:$x$$Mn^{2+}$/SIL, and CaZnOS:$x$$Mn^{2+}$/RTV-2 composite devices.

Figure S8: Normalized ML spectra of CaZnOS:$x$$Mn^{2+}$ with varied Mn^{2+} concentration.

Figure S9: Comparison of ML spectra between CaZnOS:0.03Mn^{2+} and commercially available ZnS:Cu^{+} driven by 10 N.

Figure S10: ML spectra of CaZnOS:0.03Mn^{2+}/PU, CaZnOS:0.03Mn^{2+}/SIL, and CaZnOS:0.03Mn^{2+}/RTV-2 subjected to varying dynamic loads.

1. Experimental Section

1.1 Materials

$CaCO_3$ (≥ 99.99%), ZnS (≥ 99.99%), $(CH_3COO)_2Mn\cdot4H_2O$ (≥ 99.9%), and C_2H_5OH (≥ 95%) were purchased from Adamas-Beta (Shanghai, China), Aladdin (Shanghai, China), Sinopharm (Beijing, China), and Adamas-Beta (Shanghai, China) company, respectively. Polydimethylsiloxane (PDMS, 184 Silicone Elastomer Kit), polyurethane (PU, LN-3002), silicone gel (SIL, T00), hydrophobic room-temperature vulcanized silicone rubber (RTV-2, 601) and optical epoxy resin (SpeciFix-40) were acquired from Dow Corning (Michigan, America), Leini (Shenzhen, China), Guoyuan (Shenzhen, China), WACKER (Munich, Germany) and Struers (Ballerup, Denmark) company, respectively. The commercial phosphor $ZnS:Cu^+$ (D502CT) was obtained from Keyan (Shanghai, China) company.

1.2 Preparation of $CaZnOS:Mn^{2+}$ powder

$CaZnOS:Mn^{2+}$ powder was synthesized using the conventional high-temperature solid-state method. Stoichiometric amounts of reactants were ground in an agate mortar after adding an appropriate volume of absolute ethanol to aid dispersion. The mixture was then homogenized, dried at 60 °C for 4 hours, and placed in an alumina crucible. Subsequently, it was calcined in a horizontal tube furnace at 1100 °C for 3 hours under an argon flow (100 mL min^{-1}) to serve as a protective atmosphere. The samples were allowed to cool to room temperature naturally, ground again in an agate mortar to achieve a uniform $CaZnOS:Mn^{2+}$ composition.

1.3 Preparation of $CaZnOS:Mn^{2+}$/polymer composite devices

Four polymer composite devices containing $CaZnOS:Mn^{2+}$ are prepared to measure the ML spectrum under mechanical stimuli. To create composite film containing $CaZnOS:Mn^{2+}$ (40 mm long, 40 mm wide, and 0.5 mm thick), first incorporate pre-prepared $CaZnOS:Mn^{2+}$ microparticles (0.3 g) into commercial PDMS/PU/SIL/RTV-2 (0.1 g), respectively. Note that commercial polymers mentioned above consist of precursor A and curing agent B. After thorough mixing, place the mixture between two pieces of polyethylene terephthalate films. Then, cure the blend at 70 °C for 5 hours in a drying oven to solidify the composite film. Remove the film and plasticize it at 110 °C three times to obtain the film required for testing. This process ensures the proper embedding of the microparticles within the polymer composite matrix, creating a uniform composite film suitable for optical characterization. According to the different properties of selected polymers, the ratio of PDMS/PU/SIL/RTV-2 precursor A to curing agent B is 10: 1, 1: 1, 1: 1, and 9: 1, respectively.

1.4 Preparation of $CaZnOS:Mn^{2+}$/polymer epoxy resin block

A cylindrical block containing $CaZnOS:Mn^{2+}$ is prepared to evaluate its ML repeatability when subject to cycle mechanical stimuli. To craft a cylindrical block containing $CaZnOS:Mn^{2+}$ (25 mm in diameter, 19 mm in thickness), begin by incorporating as-prepared $CaZnOS:Mn^{2+}$ microparticles into components A and B of a commercial optical epoxy resin in a weight of 1.5000 g, 5.9524 g and 2.3810 g, respectively. After thorough mixing, cure the blend at 60 °C for 24 hours in a drying oven to solidify the epoxy resin block. This process ensures the proper

embedding of the microparticles within the resin matrix, creating a uniform cylindrical block suitable for optical characterization.

2. Characterizations

The powder XRD patterns of prepared samples were collected using a powder X-ray diffractometer (Rigaku, Ultima IV) with Cu Kα radiation (λ = 1.54178 Å, 40 kV, and 20 mA). Morphological information and element distribution of the samples were analyzed using a scanning electron microscope (Zeiss Gemini 300) and an energy dispersion spectrometer (Oxford Xplore). The microstructure was characterized by a transmission electron microscopy (JEOL, JEM2100F). The EPR spectrum was characterized using a Bruker EMX plus electron paramagnetic resonance spectrometer at a frequency of 9.85 GHz. XPS spectra were obtained with a Thermo Kalpha X-ray photoelectron spectrometer. Diffuse reflectance spectra (DRS) were characterized using a Shimadzu UV-3600 ultraviolet-visible spectrophotometer over a wavelength range of 200 ~ 800 nm, with $BaSO_4$ powder serving as the standard reference. PL and PLE spectra were measured on a Hitachi F-4700 fluorescence spectrometer. The Quantum efficiency (QE) was measured by an integrating sphere system connected to Edinburgh FLS1000 spectrometer. ML spectra were measured with a custom-built system comprising an optical fiber spectrometer (Ocean Optics, QE pro) and an apparatus designed to apply and measure mechanical forces, with the photos present in Figure S5. Prior to testing, the sample was pre-irradiated for 5 minutes with an ultraviolet flashlight (275 nm, 5 W). For thermoluminescence (TL) measurement, sample was preirradiated with a 254 and a 365 nm light for 5 min and recorded at 30 s after the cessation of irradiation. TL curves were collected using a thermoluminescent dosimeter (Guangzhou Radiation Science and Technology, SL08) over a temperature range from room temperature to 400 °C at a heating rate of 1 °C s^{-1}. The data of mechanoluminescence repeatability and degradability were collected by a Hamamatsu C13796 photon counter by applied cyclic pressures to the prepared CaZnOS/epoxy resin block with a universal testing machine (Shimadzu, AGS-X). The stress range of the mechanoluminescence cyclic test is 0 ~ 5000 N, the compression rate is 10 mm·min^{-1}, and the number of cycles is 5 times. During the test, an ultraviolet flashlight (365 nm, 5 W) was used to irradiate the sample for 5 min. The repeatability data of the composite film were measured using an electromechanical universal testing machine (MTS SYSTEMS, C44.104). The tensile strain was set at 30%, the stretching speed was 300 mm min^{-1}, and the number of cycles was 250. All photos were taken using a Huawei Mate 30 mobile device. Unless otherwise specified, all measurements were conducted at room temperature.

3. Figures and Tables

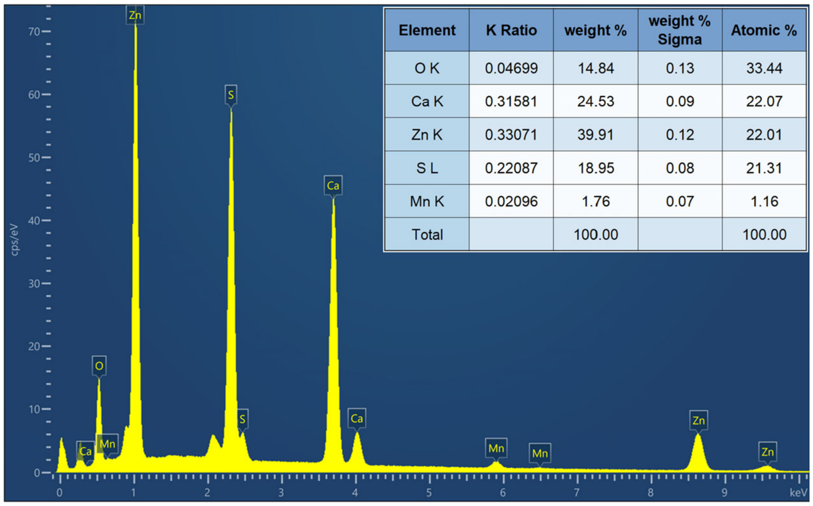

Element	K Ratio	weight %	weight % Sigma	Atomic %
O K	0.04699	14.84	0.13	33.44
Ca K	0.31581	24.53	0.09	22.07
Zn K	0.33071	39.91	0.12	22.01
S L	0.22087	18.95	0.08	21.31
Mn K	0.02096	1.76	0.07	1.16
Total		100.00		100.00

Figure S1. EDS spectrum and elemental composition of CaZnOS:0.05Mn^{2+}.

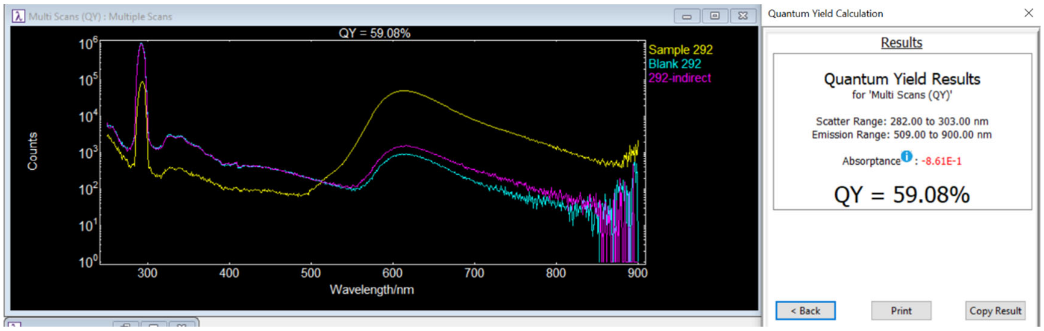

Figure S2. The quantum efficiency of CaZnOS:0.01Mn^{2+}.

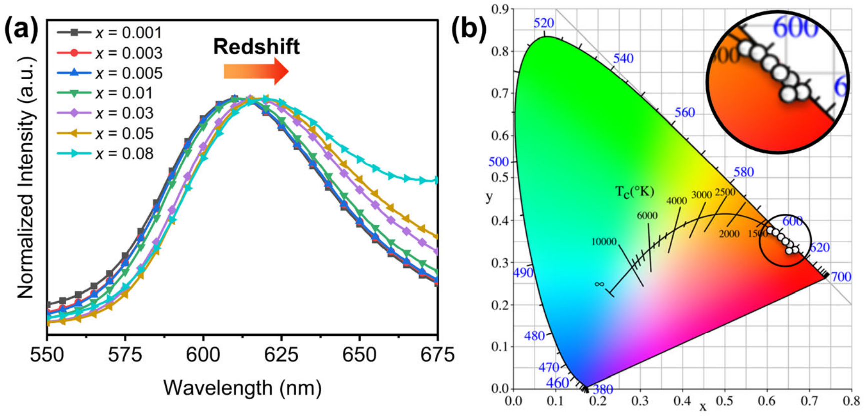

Figure S3. (a) Normalized PL spectra; (b) CIE coordinates of CaZnOS:xMn^{2+} with varied Mn^{2+} concentration.

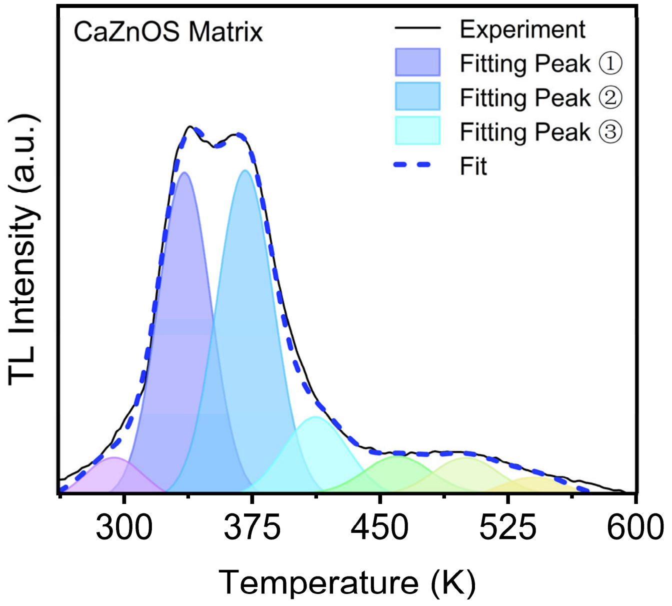

Figure S4. Fitting result of the TL spectrum of CaZnOS matrix.

Tbale S1. Fitting result parameters of the TL spectra of CaZnOS:0.03Mn^{2+} and CaZnOS matrix.

***x* = 0.03**	**Trap depth (eV)**	**Temperature (K)**	**Area**	**ω**
Trap ①	0.807	334.4	2640	39
Trap ②	0.867	370.2	1269	46
Trap ③	0.949	424	236	55
***x* = 0**	**Trap depth (eV)**	**Temperature (K)**	**Area**	**ω**
Trap ①	0.873	335.2	1335	36.4
Trap ②	0.999	370.8	1238	39
Trap ③	1.079	412.8	175	44.7

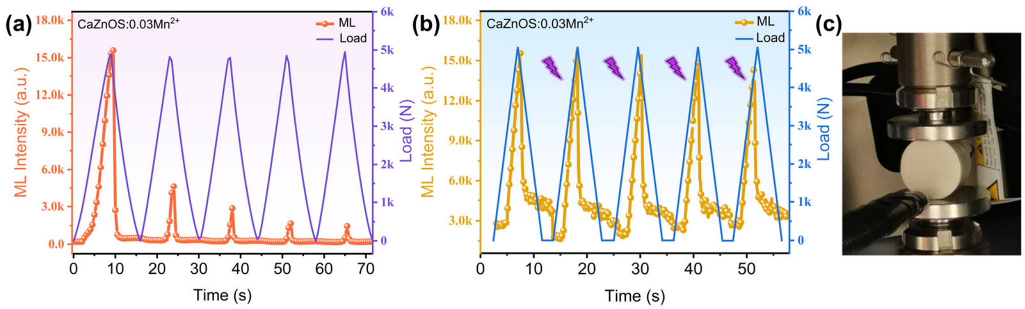

Figure S5. (a) ML degradability and (b) repeatability of CaZnOS:0.03Mn^{2+}/epoxy resin composite. (c) The photo of the testing sample: CaZnOS:0.03Mn^{2+}/epoxy resin composite block.

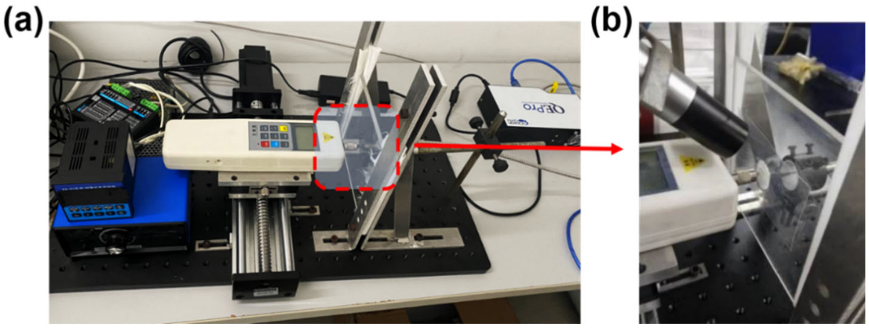

Figure S6. The custom-built system for collecting mechanoluminescence spectrum.

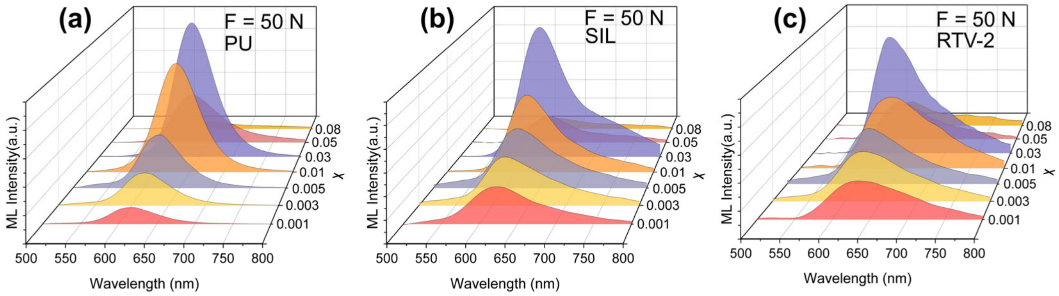

Figure S7. ML spectra of CaZnOS:$x$$Mn^{2+}$/PU, CaZnOS:$x$$Mn^{2+}$/SIL, and CaZnOS:$x$$Mn^{2+}$/RTV-2 composite devices.

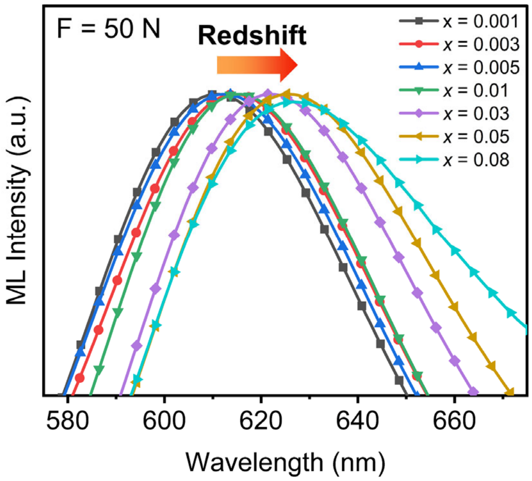

Figure S8. Normalized ML spectra of CaZnOS:xMn^{2+} with varied Mn^{2+} concentration.

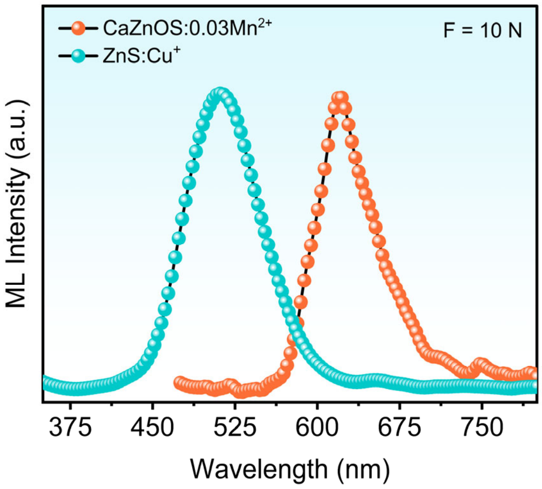

Figure S9. The ML spectra of CaZnOS:0.03Mn^{2+}/PDMS and commercially available ZnS:Cu^{+}/PDMS, , both driven by a force of 10 N.

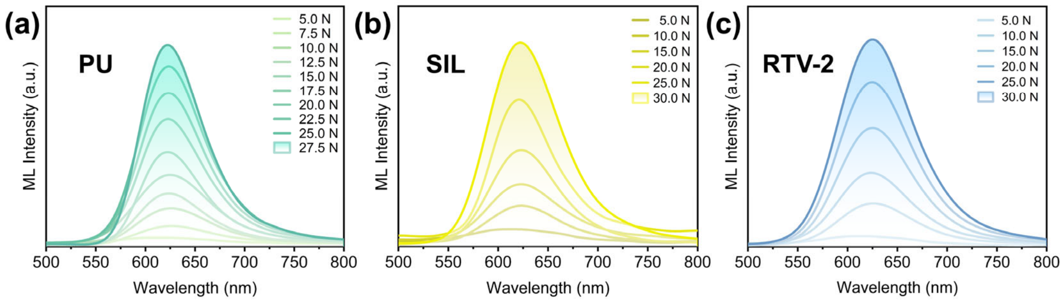

Figure S10. ML spectra of CaZnOS:0.03Mn^{2+}/PU, CaZnOS:0.03Mn^{2+}/SIL, and CaZnOS:0.03Mn^{2+}/RTV-2 subjected to varying dynamic loads.
